# COVID-19 Vaccination within the Context of Reactogenicity and Immunogenicity of ChAdOx1 Vaccine Administered to Teachers in Poland

**DOI:** 10.3390/ijerph19053111

**Published:** 2022-03-06

**Authors:** Maria Ganczak, Marcin Korzeń, Ewa Sobieraj, Jakub Goławski, Oskar Pasek, Daniel Biesiada

**Affiliations:** 1 Department of Infectious Diseases, University of Zielona Gora, 65-417 Zielona Gora, Poland; 2 Department of Methods of Artificial Intelligence and Applied Mathematics, West Pomeranian Institute of Technology, 71-210 Szczecin, Poland; mkorzen@wi.zut.edu.pl; 3 Student Research Group, University of Zielona Gora, 65-417 Zielona Gora, Poland; ewa.sobieraj@gmail.com (E.S.); jakubgolawski2@gmail.com (J.G.); oskar.pasek2@gmail.com (O.P.); 4 Primary Care Clinic “Lancet”, 73-240 Bierzwnik, Poland; d.biesiada@gmail.com

**Keywords:** ChAdOx1 vaccine, teachers, adverse effects, immunogenicity, determinants

## Abstract

In February 2021, Polish teachers were offered the ChAdOx1-S vaccine as a priority group. However, there have been concerns among educators regarding the efficacy of this vaccine, as compared to the other types of vaccines (e.g., mRNA). The objective of this study was to investigate the reactogenicity and the immunogenicity of this vaccine. Participants, specifically teachers, were invited for serological testing ≥ 4 weeks post-vaccination. Antibodies against the receptor-binding domain (RBD) were measured. Of the 192 participants, the mean age was 50.5 ± 8.3 years and the mean (range) dosing interval was 69.6 ± (25–111) days. Adverse reactions included feeling feverish (44.8%), headache (41.7%), malaise/chills (38.0%), and injection-site tenderness (37.5%); these were reported more frequently after the first dose (84.9%). Fewer males than females (54.8% vs. 80.1%) and fewer older participants (65.7% vs. 90.4%) reported side effects (*p* < 0.002; *p* < 0.0001, respectively). All participants presented detectable anti-RBD IgG; the median (range) reading was 525.0 BAU/mL (20.6–5680.0); 1008.02 BAU/mL (115.3–5680.0) in those with prior SARS-CoV-2 infection; and 381.42 BAU/mL (20.6–3108.8) in those without (*p* = 0.001). In 27.6%, the anti-RBD IgG level was >500 BAU/mL. A multivariate logistic regression revealed that previous infection and longer dose intervals were predictors of higher immunologic responses (*p* < 0.0001; *p* = 0.01, respectively). The results demonstrated good tolerability and immunogenicity of the ChAdOx1-S vaccine. Our study justified the longer dose interval to enhance a higher antibody response. Our findings may also support the prioritization of uninfected individuals in regions where COVID-19 vaccine-sparing strategies are required.

## 1. Introduction

Multiple coronavirus disease 2019 (COVID-19) vaccines have been developed globally as the most effective preventive method to combat the severe acute respiratory syndrome coronavirus 2 (SARS-CoV-2) pandemic [1]. Poland, along with many other European Union (EU) countries, started its National COVID-19 Vaccination Program on 27 December 2020 with the introduction of the Pfizer-BioNTech BNT162b2 vaccine. The vaccine regimen was gradually expanded with three other products approved for use including Moderna mRNA-1273, Oxford-AstraZeneca ChAdOx1 nCoV-19, and Johnson and Johnson/Jansen Ad26.COV2.S [2].

COVID-19 vaccinations were rolled out in phases. In the first phase, all EU countries started vaccinating priority groups that were determined based on their higher risk of developing severe disease, in addition to healthcare and other front-line workers. Poland prioritized elderly people (≥80 years old), residents and personnel in long-term care facilities, healthcare workers (HCWs), and essential public service workers such as those working in educational institutions [2,3]. Of note, Poland was one of the first countries to recommend the vaccination of teachers in accordance with the recommendation by United Nations Educational, Scientific, and Cultural Organization (UNESCO) [4].

As of 12 February 2021, the ChAdOx1 nCoV-19 vaccine was provided for the voluntary immunization of Polish teachers [5]. The vaccine was developed at the University of Oxford and produced by AstraZeneca, and it was quickly adopted worldwide. It employs a chimpanzee adenoviral vector vaccine with a full-length SARS-CoV-2 spike insert. The genetic sequences contained in the adenovirus encode the synthesis of the SARS-CoV-2 coronavirus surface protein S [6]. The assessment of vaccine-generated immune responses to SARS-CoV-2 spike antigens has mostly focused on the development of antibodies targeting the S1 domain of the viral spike protein. More than 99% of participants in an Randomized Control Trial (RCT) conducted by Folegatti et al. had neutralizing antibody responses by 14 days after the second dose [7]. Eyre et al. also reported that vaccination with the ChAdOx1 nCoV-19 vaccine led to detectable anti-spike antibodies in nearly all adult HCWs [8]. A key benefit of the vaccine was that anti-S IgG titers were higher than for natural infection [9]. Anti-spike antibody titers, associated with neutralizing activity, provided a potential surrogate marker of protection [7,8,9,10,11]; higher levels of immune markers were correlated with a reduced risk of symptomatic infection.

Higher S-binding antibodies were observed with increasing dose intervals [12,13]. In Poland, the vaccination schedule was initially adopted in accordance with the recommendations of the producer, with two doses of the vaccine administered at an interval of 10–12 weeks apart [6]. On 17 May 2021, following governmental regulations, the interval between doses was reduced to 35 days [14].

The governmental recommendations to limit the vaccines provided to Polish teachers solely to the ChAdOx1 nCoV-19 vaccine was met with a wave of criticism and dissatisfaction, as the ChAdOx1 was perceived as inferior to messenger ribonucleic acid (mRNA) vaccines. The most common argument was its questionable effectiveness [5,15]; the overall vaccine efficacy (VE) of 66.7% against symptomatic infection and 27.3% against asymptomatic infection was reported as a result of four randomized control trials (RCTs) in relation to ChAdOx1 [12]. Additionally, the Polish government’s decision to reduce the interval between doses, which had not been evidence-based, left some confused and questioning its potential impact on the vaccine immunogenicity. There were also emerging reports concerning its side effects, such as flu-like symptoms accompanied by high fever and muscle pain as well as blood clots, which were listed as a very rare side effect of the vaccine [16,17,18,19,20].

Understanding the time-dependent dynamics of post-vaccine anti-spike antibody measurements and assessing how they differ between individuals (e.g., by age, sex, body mass index (BMI), comorbidities, etc.) has also been an increasing concern [8,12,17,21].

Universal teacher immunization remains crucial in order to ensure the continuity of education. To achieve this, it is necessary to build vaccine trust among this professional group through wide-ranging information campaigns as well as continuous scientific research to better determine the safety, tolerability, immunogenicity, and effectiveness of COVID-19 vaccines.

Considering the need for practical evidence for the ChAdOx1 nCoV-19 vaccine and since such data have not yet been assessed in Polish adults, specifically the working-age population, this study assessed the reported adverse events following immunization and measured the anti-spike IgG responses in teachers following two vaccine doses. The determinants of SARS-CoV-2 anti-spike IgG responses were evaluated, including previous infection.

## 2. Materials and Methods

### 2.1. Population and Setting

Post-vaccine antibody responses were studied between June–July 2021 in teachers recruited from primary, secondary, and high schools via local teacher networks. Convenience sampling design was adapted to recruit teachers with a goal of 200 participants. All schools were located in the capitals of two Polish provinces: Zielona Gora and Szczecin. The inclusion criteria included being employed as a teacher and being immunized with two doses of the ChAdOx1 nCoV-19 vaccine at least four weeks prior to the survey. The last criterion was based on the results of an RCT of the ChAdOx1 nCoV-19 vaccine, which had shown that antibodies against the receptor-binding domain (RBD) after a second dose had peaked by day 28 in the vaccine recipient group and had remained elevated to day 56 [7].

Teachers contacted the research team directly via a dedicated phone line if they wanted to participate. Following initial contact with the study team, participants were then informed regarding the phlebotomy time and the health care facility address. At the phlebotomy time point, the participant information sheet was given to each teacher, and written consent was obtained.

### 2.2. Study Instrument

A short questionnaire was developed by the authors after an intensive literature review [6,7,8,11,12,13,16,17,18,19,20,21]; the opinions of a panel of three experts (an immunologist, an epidemiologist, and an infectious disease specialist) were also taken into consideration. In order to guarantee clarity, validity of content, as well as internal consistency, the original version of the questionnaire was distributed to 15 respondents prior to initiating the survey (pilot phase). Following the review of the questions raised by the educators, the study instrument was adjusted by the research team.

The questions concerned sociodemographic data including age, sex, school location, and core health risk factors (body-mass index (BMI), smoking status, and presence of comorbidities including type 2 diabetes, cancer, heart/lung/kidney disease). Participants were then asked to record the date of the ChAdOx1 nCoV-19 vaccine administration and dosing interval; this was then reviewed by a research team member via the national COVID-19 vaccination database. Participants were asked whether they had experienced adverse effects, including both systemic and local effects. Systemic side effects included symptoms such as fatigue, malaise, headache, chills, fever, arthralgia, myalgia, nausea, and diarrhea; local side effects included injection site pain, tenderness, redness, and swelling [12,17]. Participants could also tick “other adverse effects” or “no symptoms”. Data on any previous SARS-CoV-2 infection were available to the research team from the national patients’ database. As some patients had not been tested for SARS-CoV-2 infection despite a medical history of COVID-19-like symptoms and/or contacts with infected patients, the previous infections reported by participants in the study questionnaires were also considered while assessing individuals previously infected with SARS-CoV-2.

The questionnaire was submitted to the participants before the phlebotomy.

### 2.3. Laboratory Assays

Blood samples were collected by a qualified nurse or physician. Samples (5 mL) were centrifuged (15 min/4500 rpm.), stored at 4–8 °C, and then transported to the Synevo laboratory in Cracow, Poland where they were tested. Briefly, post vaccination anti-spike IgG responses were assessed using the Abbott SARS-CoV-2 IgG II Quant antibody assay, an automated, two-step chemiluminescent microparticle immunoassay (CMIA) targeting the spike RBD. The assay was used for the qualitative and quantitative assessment of IgG antibodies to SARS-CoV-2 in human serum and plasma on the ARCHITECT i System. The assay cut-off was ≥7.1 BAU/mL, as reported by the manufacturer. The sensitivity (based on ≥14-day post-positive reverse transcription-Polymerase Chain Reaction (PCR) samples) and the specificity of the Abbott anti-nucleocapsid assay had been previously evaluated as 98.3% (90.6–100.0%) and 99.5% (97.1–100%), respectively [22].

Each study participant was given a code number that was recorded both on the questionnaire and on the test tube. After 11 July 2021, the participants were able to obtain information about their post-vaccination serological test results.

### 2.4. Vaccination Immunogenicity Assessment

On the basis of the results obtained after sero-testing, geometric mean titers (GMTs) were calculated at ≥4 weeks after vaccination to assess the immunogenicity of the ChAdOx1-S vaccine. Furthermore, we calculated a fraction of the participants who achieved an anti-RBD IgG response > 500BAU/mL, the threshold associated with a VE of 80% against symptomatic COVID-19 infection [22].

### 2.5. Statistical Analysis

Data were analyzed using a customized program, STATISTI-CA PL, version 12.5 (StatSoft, Cracow, Poland, 2016). Categorical data were presented both as frequencies with percentages as well as continuous data with means and ranges. The participants were grouped into those with evidence of prior infection (i.e., those who reported having any positive anti-spike or anti-nucleocapsid antibody test or positive PCR prior to the first or second vaccination and those who reported having COVID-19 without confirmation by any diagnostic test) and those without (including participants with no previous serology or PCR testing). Categorical variables were compared using the chi-squared test while continuous variables were compared using the Student’s t-test. Correlations were calculated using the standard Pearson’s correlation coefficient. The occurrence of adverse effects was studied for the first and the second dose of the ChAdOx1 nCoV-19 vaccine. To assess the determinants of the occurrence of adverse effects, we used the following strata: age (≤55 years vs. >55 years), sex, previous SARS-CoV-2 infection (binary variable of yes/no), smoking status (binary variable of current/previous smoker vs. non-smoker), obesity (BMI < 30 kg/m^2^ vs. ≥30 kg/m^2^), and comorbidities (binary variable of with/without comorbidities).

The proportions of anti-RBD-positive participants were estimated by checking an anti-RBD IgG antibody titer at least four weeks after the second dose. The primary endpoint, anti-RBD IgG titer, was analyzed using GMT. Multivariate logistic regression was applied to determine the predictors of immunogenicity. We modelled quantitative IgG antibody titers using several multivariate logistic regression models. The preliminary model took into consideration eight variables: age, gender, previous SARS-CoV-2 infection, obesity, comorbidities, and smoking status, as well as dosing interval and time of evaluation. All models were reduced by the use of the stepwise backward elimination method [23]. Non-standardized regression coefficients in the regression model were used to evaluate any changes in the model. The regression results were presented together with 95% confidence intervals (CIs). A *p*-value was statistically significant if ≤0.05.

## 3. Results

Overall, 200 participants were invited to participate, of whom 8 were disqualified due to incorrect information regarding their vaccination status. A total of 192 participants were tested for anti-RBD SARS-CoV-2 IgG. Their demographic details and SARS-CoV-2 infection status prior to immunization are given in Table 1.

The mean age of the participants was 50.5 years, with a standard deviation (SD) ± 8.3 and range 27–67 years—16.1% were male. Regarding BMI, 38% were overweight and 13% were obese. Smoking at the time of vaccination and/or in the past was reported by 12% of the participants while 75% reported that they had never smoked. Almost a half of the participants (45.4%) reported comorbidities, mainly diabetes (9.9%), followed by cardiovascular disease (6.8%) and respiratory tract disease (4.8%). Previous SARS-CoV-2 infection was reported by 49 (25.5%) participants. Of those, 27 had an infection that had been confirmed by a PCR or rapid antigen test; the rest reported having had COVID-19 that had not been confirmed by a test.

More than a half of participants (58.3%) reported they would change the product for another vaccine if there were an opportunity; 16.1% would not and 25.5% were unsure. An mRNA vaccine was the most common option chosen (Pfizer-BioNTech BNT162b2 Comirnaty, 88.4% and Moderna mRNA-1273 Spikevax, 7.1%); another vector vaccine such as Janssen COVID-19 Ad26.Cov-2.S vaccine was the rarest choice (2.5%).

### 3.1. Adverse Effects

Local and/or systemic reactions after receiving the first or second dose were reported by 79.2% (n = 152) of the participants. Of those, 129 participants reported the adverse effects as more expressed after the first dose; 6 reported after after the second dose; 10 were the same after the first and the second dose; and 7 participants were not sure. Among vaccinated participants, 53.6% reported one or more local adverse effects and 72.9% indicated having one or more systemic adverse effects. The most common local reaction after receiving the vaccine was tenderness at the injection site at 37.5%, followed by experiencing injection-site pain at 32.3%. When reviewing the number of reports on systemic side effects, the most common were feeling feverish (44.8%), followed by headache (41.7%), malaise (38.0%), chills (38.0%), and fatigue (36.5%).

Females were more likely to report adverse effects than men (129/161; 80.1% vs. 17/31; 54.8%, *p* < 0.002). Local and systemic reactions were more common in participants aged < 50 years, as compared to older participants (75/83; 90.4% vs. 71/108; 65.7%, *p* < 0.0001). Among the participants that reported experiencing side effects, the difference between those who had been previously infected with SARS-CoV-2 versus those who had not was not significant (35/50, 79.5% vs. 111/151, 75.5%; *p* = 0.58).

### 3.2. Dosing Interval, Time of Evaluation

The mean (range) dosing interval was 69.6 ± 10.4 (25–111) days and only 4 participants reported a dosing interval shorter than 7 weeks (i.e., 25, 26, 45, and 46 days). A total of 32.8% received their second dose between the 7th and 10th week after the first dose and 65.1% received their second dose ≥ 10 weeks after the first. The mean time between the second dose and a serological test was 50 ± 9.2 days (28–95 days); in 74.0% of the participants, the time between the second dose and a serological test was up to 56 days.

### 3.3. Antibody Response

By ≥4 weeks after the second dose, all participants presented detectable levels of anti-RBD SARS-CoV-2 IgG. Median anti-RBD IgG was 525.0 BAU/mL (20.6–5680.0). In eight (4.2%) participants (5 males and 3 females; aged 43–66 years; BMI, 22.7–30.2 kg/m^2^; time range between the second dose and a serological test, 37–80 days; 2 reported comorbidities; none reported previous infection), the anti-RBD IgG reading was below 50.0 BAU/mL (range 20.6–43.5). More than one quarter of the participants (53/192; 27.6%) presented anti-RBD IgG > 500 BAU/mL.

**Figure 1:**
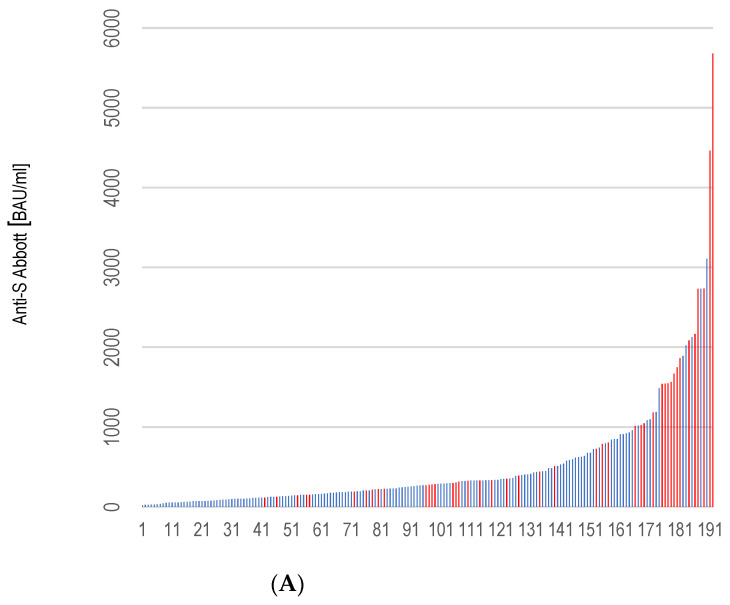

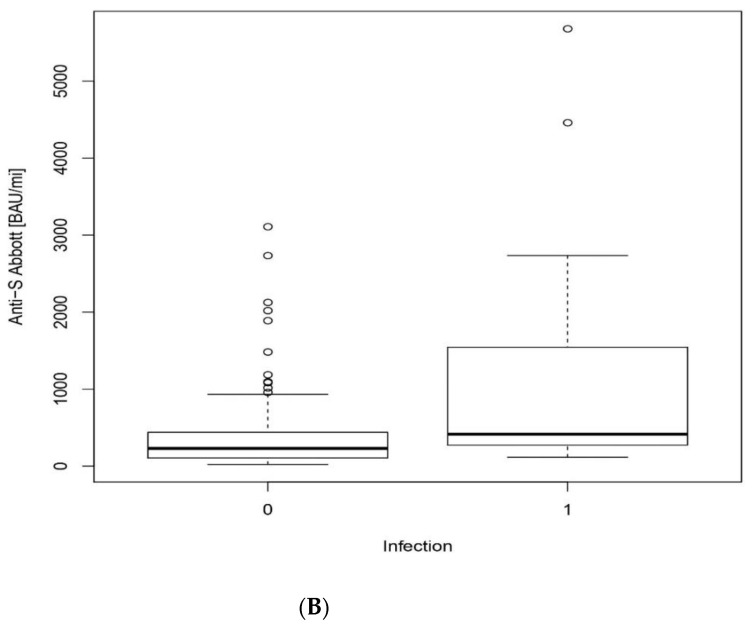
Antibody responses among 192 participants following second vaccination with ChAdOx1 with prior infection. Data are shown as a bar chart of a cohort where participants with previous SARS-CoV-2 infection are shown in red on the bar chart (**A**) and as a box plot that displays the median values (**B**) with the interquartile range, and ±1.5-fold the interquartile range from the first and third quartile (lower and upper whiskers).

As expected, those with previous infections developed substantially higher titers of anti-RBD IgG. The median (range) anti-RBD IgG reading was 1008.02 (115.3–5680.0) BAU/mL in participants with prior infection and 381.42 BAU/mL (20.6–3108.8) in those without infection (*p* = 0.001). Figure 1 illustrates the antibody responses following a two-dose vaccination course with ChAdOx1 nCoV-19 and with prior infection. 

### 3.4. Correlation of the Rate of Anti-S Antibody Titers and Selected Variables

A positive, statistically significant correlation was observed between the dose interval and age (Pearson’s correlation r = 0.207, 95% CI: 0.065 and 0.341; *p* = 0.006) as well as the dose interval and anti-RBD titers (r = 0.144, 95% CI: 0.002 and 0.280; *p* = 0.046). A negative, statistically significant correlation was detected regarding the days between receiving the second dose and performing a serological test (r = −0.198, 95% CI: −0.322 and −0.048; *p* = 0.009) and the anti-RBD titers.

Table 2 presents the anti-RBD IgG titers after the second dose of vaccine by selected variables in previously uninfected and infected participants.

Significantly higher anti-RBD IgG titers were reported among older participants (≥60 years old) with previous SARS-CoV-2 infection as compared to younger participants (*p* < 0.0001), and, among those, higher titers were reported in participants who were obese (≥30 kg/m^2^) as compared to those who reported a BMI < 30 kg/m^2^ (*p* = 0.04).

Regarding participants previously infected with SARS-CoV-2, as well as those uninfected, no statistically significant differences were observed concerning sex, comorbidities, smoking status, and anti-RBD IgG titers. Among previously uninfected participants, there were no statistically significant differences regarding age, BMI, and anti-RBD IgG titers.

### 3.5. Predictors of Immunogenicity

Multiple logistic regression analyses regarding the association of immunogenicity (measured by the level of anti-RBD IgG titers) with the selected variables revealed that previous infection with SARS-CoV-2 and longer dose intervals were independent positive predictors of a higher immunologic response (*p* < 0.0001 and *p* = 0.01, respectively); Table 3.

## 4. Discussion

### 4.1. Dosing Interval, Time of Evaluation

The mean dosing interval was 69 days, with a range of 25–111 days; only 1% of participants reported a shortened interval (i.e., less than 5 weeks) between ChAdOx1 nCoV-19 doses. This indicated that the vast majority of the study participants had not followed the Polish government’s recommendation of 35 days between doses [5]. This regulation was in opposition to the policies issued by the United Kingdom and several other countries that suggested an extended interval, where the second dose of the ChAdOx1 nCoV-19 vaccine was delayed for 10–12 weeks following the first dose [6,24].

### 4.2. Frequencies and Intensity of Adverse Reactions

We found that systemic adverse effects affected almost three out of four participants and local side effects affected more than every second teacher. Similar results regarding reported systemic adverse events (71.6%) were found in an RCT concerning the ChAdOx1 nCoV-19 vaccine [19]. However, more participants in the RCT group than in our study reported local adverse events (74.1%). The reactogenicity of the ChAdOx1 nCoV-19 vaccine in participants of up to 67 years of age was also comparable to that reported in other RCTs [7,12,17,18] and was listed in the product information [6], and was similar to the anecdotal observations of other authors who conducted research outside of clinical trials [20,21].

Furthermore, the adverse effects observed in this study were similar in nature to those previously reported [17,19,20]. Severe systemic allergic reactions following immunization, such as anaphylactic shock, were not reported by the study participants. However, the small sample size reduced the likelihood to detect such adverse events. Recent data indicated that an incidence of anaphylactic shock was very infrequent (1 per 200,000–1 per million doses) [25]. Although not found in our study, there were also some reports of very rare side effects such as arterial events, venous thromboembolism, thrombocytopenia, and bleeding after vaccination with ChAdOx1-S [26,27,28].

Fewer adverse events among the studied participants were found after the second vaccination than after the initial vaccination. Similar results have been found in other studies that have assessed the safety of the ChAdOx1 nCoV-19 vaccine [17,19,20]. Of note, some other studies found that individuals vaccinated with the ChAdOx1 nCoV-19 vaccine were more likely to experience systemic side effects than those who had been given the BNT162b2 vaccine [29].

Side effects were more prevalent in participants aged < 50 years than in older participants. Our results provided evidence to support other studies, both RCTs and those conducted in the community, of a lower occurrence of side effects in older individuals [17,19,20,29]. We found that adverse effects were less common in males versus females, which was also consistent with previous studies [20].

Although local and systemic side effects have been reported to be higher in individuals previously infected with SARS-CoV-2 than in those without any known past infections, for both BNT162b2 and ChAdOx1 nCoV-19 vaccines [20,30,31,32], this was not observed in our study.

### 4.3. Anti-RBD Antibody Titers

All participants presented detectable anti-RBD IgG antibodies ≥ 4 weeks after receiving two doses of the ChAdOx1 vaccine; however, 4.2% of the participants (and none of these reporting any previous SARS-CoV-2 infection) were low-responders. Some other studies have reported similar rates of low response among participants; however, in contrast with our results, this has been independently associated with several long-term health conditions [21].

Data from a randomized efficacy trial of the ChAdOx1 nCoV-19 vaccine determined that the antibody level > 506 BAU/mL was associated with 80% VE against symptomatic infection with the alpha (B.1.1.7) variant of SARS-CoV-2 [33]. According to these results, approximately one-fourth of participants from our study could gain such high protection. If so, our findings showed that although the ChAdOx1 nCoV-19 vaccine was highly effective for the induction of RBD-specific immune responses in a working-age population, such as teachers, the afforded thresholds for immune markers, especially in naïve individuals, might not be associated with high protection against symptomatic COVID-19.

However, it was suggested that despite modest levels of neutralizing antibody, other mechanisms might be at play as co-correlates of protection [29]. Furthermore, considerable variations can exist between patients. Infections can be still observed at high antibody levels, suggesting that a definitive individual threshold of protection is difficult to determine. Although observations made by Feng et al. [33] indicated that the reduced neutralizing capacity against the various variants of SARS-CoV-2 may have reduced protection against initial infection (and perhaps mild, symptomatic COVID-19), protection against more severe forms of disease, such as those causing hospitalization or death, had been maintained [12,29].

Previous SARS-CoV-2 infection was the biggest positive predictor of the magnitude of quantitative antibody response post-second dose, and median readings regarding anti-RBD IgG were 2.6-fold higher in individuals with prior infection, as compared to those without prior infection (Figure 1). This had also been reported by other authors who had explored this trend of the ChAdOx1 nCoV-19 vaccine [13,21], as well as other vaccines [21,34,35,36]. For example, a study conducted among Israeli healthcare workers (21 days post-first-dose of the BNT162b2 mRNA COVID-19 vaccine) found that those with prior infection had antibody titers one order of magnitude higher than naïve individuals [32]. These findings suggested prioritization for uninfected persons in regions where COVID-19 vaccine-sparing strategies were required [35,36]; such prioritization may also be applied for the third vaccination in previously infected healthy individuals.

Concerning our second main finding, longer dose intervals led to greater immune responses to the ChAdOx1 nCoV-19 vaccine; this had also been confirmed by other studies [12,13]. An analysis of RCTs found that efficacy was higher if the second ChAdOx1 nCoV-19 dose was received 8–11 weeks after the first. It was further increased if participants received the second dose more than 11 weeks after the first [12]. Furthermore, longer prime-boost intervals that yielded higher efficacies in clinical trials positively correlated with GMTs of anti-SARS-CoV2 spike IgG-binding antibodies. A similar boost to antibody responses was found with a longer duration in other studies concerning the BNT162b2 COVID-19 vaccine [11,37].

Among demographic factors, age has been consistently reported as being associated with antibody responses after COVID-19 vaccinations due to the age-related decline in immune functions [21,38]. Two-dose mRNA-vaccine candidates have shown immunogenicity in older adults, but absolute neutralizing antibody responses in adults aged 65–85 years were lower than in those aged 18–55 years [39]. A two-dose inactivated virus vaccine also showed lower absolute neutralizing antibody titers in adults aged 60 years and older than in adults aged 18–59 years [40]. No statistically significant differences in the antibody response between age groups were found in this survey, possibly due to the defined study population (i.e., teachers at a relatively young age (mean of 50.5 years)). Some other studies have found [11,12,17] that two vaccine doses achieved high responses across all age groups, which our findings confirmed. Additional studies on post-vaccination antibody responses in different age groups would be of value.

Findings regarding gender-related antibody responses to COVID-19 vaccines are inconsistent. For example, Jalkanen et al. found that, after receiving the second dose of the BNT162b2 mRNA COVID-19 vaccine, female vaccinees had slightly higher neutralization titers than males, although the anti-S1 IgG antibody levels remained at the same level [41]. However, other authors who conducted larger population studies reported that females generated stronger humoral immunity than males [21,42]. For example, data of humoral immune response to vaccination in medical staff, tested before and 15 and 90 days following vaccination, showed that women presented higher antibody levels than men, independent of age [43]. Previous studies found that differences between the sexes in antibody responses were more marked above 60 years of age [10].

In our study, no association was found between sex and ChAdOx1 vaccine antibody response. This may have been due to the small sample size and relatively small representation of males in the sample. This echoed the demographic profile of Polish teachers, where males comprised only 17.8% [44]. The problem of under representativeness of the male gender was also reported by other authors who conducted studies related to the reactogenicity and immunogenicity of COVID-19 vaccines. For instance, male vaccinees were underrepresented in the Finish study [41] of healthcare workers, comprising only 17% of the vaccinees. Similarly, Oliviera-Silva et al. presented data of humoral immune response to vaccination in Portuguese healthcare workers, of which only 24% were males [43]. Therefore, regarding sex discrepancies, the results may be biased and the cohort may not be representative for the entire population of working adults.

Although many long-term health conditions, such as rheumatoid arthritis, chronic liver disease, type-2 diabetes, obesity, asthma, and hypertension, as well as taking corticosteroids and immunosuppressants, had been independently associated with low responses [21,45], we did not detect any significant differences in antibody responses regarding participants with comorbidities and healthy individuals. This may be attributable to the relatively low fraction of participants reporting any long-term health conditions.

### 4.4. Limitations

Limitations existed in this study. The sample size of our study population was relatively small, which may have hindered some of the associations. Second, self-reported data were used for variables such as BMI and comorbidities, as well as (in 45% of participants) regarding previous SARS-CoV-2 infection. This may have introduced information bias together with misclassification. The time of evaluation ranged between 4 and 13 weeks following the second immunization, introducing time as a possible bias as well. However, according to RCT results [7], anti-RBD Ig antibodies after a second dose remained elevated up to day 56. As the mean time of evaluation in our study was 50 days and 74% of the participants had a mean time between the second dose and a serological test up to 8 weeks, the study had the potential to detect elevated levels of antibodies after the second dose. Although the vast majority of participants reported adverse events as more intense after the first dose than after the second one, the frequency assessed in this study could have been slightly higher than if it were assessed separately for each dose of the vaccine. Furthermore, the T-cell response was not assessed in this study. Although the correlation between the antibody response and vaccine efficacy is high, which has suggested that the neutralizing antibody response is important [7], T-cell responses may contribute to protection from COVID-19 even in the presence of lower neutralizing antibody titers [10]. Finally, the study design (a cross-sectional study) did not allow an inference of causality [20].

## 5. Conclusions

This report demonstrated that the ChAdOx1 nCoV-19 vaccine appeared safe, well-tolerated, and immunogenic in the studied population. Although mild adverse effects affected the majority of participants, particularly after the first dose, no serious side effects were reported. Detectable anti-RBD IgG antibodies were presented by all participants. Significantly higher immunogenicity was observed in participants who reported previous SARS-CoV-2 infection. The latter finding suggested that in immunocompetent vaccine recipients with evidence of previous infection, a delay of the second dose, and possibly the third, could be considered when careful management of vaccine resources is needed. Furthermore, our study justified the longer dose interval as an important factor to enhance higher antibody responses post-vaccination with ChAdOx1 nCoV-19.

The results may help to reduce the concern among Polish educators regarding the inferior characteristics of the ChAdOx1 nCoV-19 vaccine. As the vaccine is likely to be one of the least expensive among of all currently authorized COVID-19 vaccines, addressing these concerns is critical.

## Figures and Tables

**Table 1:** Demographics of participants who received a second dose of ChAdOx1 nCoV-19; Poland, 2021, n = 192.

Variable	n	%
Sex		
Female	161	83.9
Male	31	16.1
Age (years): * 50.5 (27–67)		
27–40	17	8.9
41–50	73	38.0
>50	102	53.1
BMI kg/m^2^		
<25	90	49.2
25.0–29.9	69	37.7
≥30	23	13.1
Smoking		
current	23	12.0
quit	26	13.5
never	143	74.5
Comorbidity		
Cardiovascular disease	13	6.8
Respiratory disease	8	4.2
Diabetes	19	9.9
Other **	47	24.5
No comorbidity	105	54.6
Previous SARS-CoV-2 Infection		
Assessed by the test ***	27	14.1
Assessed by the participant	22	11.4
No infection	143	74.5

* Mean (range); ** rheumatoid arthritis, chronic liver disease, cancer, taking corticosteroids, taking immunosuppressants; *** PCR/antigen/serologic test.

**Table 2:** Anti-RBD IgG titers after the second dose of vaccine by selected variables in previously uninfected and infected participants.

Variable	Previously Uninfected			Previously Infected with SARS-CoV-2		
	GMT	N	*p*	GMT	N	*p*
Sex						
Female	347.7	121	0.51	1048.1	41	0.89
Male	413.2	24		984.7	7	
Age (years)						
<40	355.7	13	0.95	741.8	4	0.0001
40–60	347.3	120		862.5	39	
≥60	473.2	12		3053.6	4	
BMI						
<25 kg/m^2^	336.5	67	0.86	803.5	24	0.04
≥25–29.9 kg/m^2^	350.0	57		866.1	15	
≥30 kg/m^2^	462.7	15		1666.1	6	
Comorbidities *						
Yes	335.1	98	0.57	833.3	35	0.52
No	383.7	39		1150.1	7	
Current/Previous smoker						
Yes	440.5	19	0.37	942.3	36	0.30
No	536.6	128		1354.2	11	

* Rheumatoid arthritis, chronic liver disease, cancer, taking immunosuppressants, corticosteroids.

**Table 3:** Logistic regression analysis: association of anti-RBD IgG titers with selected variables; estimates, 95% confidence intervals (CIs), and *p* values; n = 192.

Variable	Estimate	95% CI	*p*
Intercept	−1173.84	−2161.24–186.45	0.02
Age	10.83	−1.79–23.64	0.09
SARS-CoV-2 infection	545.17	300.69–798.64	<0.0001
Dose interval	14.31	3.43–25.20	0.01

## Data Availability

The data underlying this article will be shared upon reasonable request to the corresponding author.
